# Supplementary material for: Psychological risk indicators of disordered eating in athletes
Source: PLoS One. 2020 May 14;15(5):e0232979. doi: 10.1371/journal.pone.0232979 (PMC7224458; doi:10.1371/journal.pone.0232979)
Supplement: Attachment — Submitted filename: Review.docx [file pone.0232979.s002.docx]

This study entitled “Psychological Risk Indicators of Disordered Eating in Athletes” aimed at assessing the applicability of the theoretical etiological model proposed by Petrie and Greenleaf (2007; 2012) in a large sample of athletes. Overall, the study addresses a gap in the current literature and I believe that it offers an interesting contribution, but would be strengthened by addressing the following concerns.

**Introduction:**

*General feedback:* The authors need to spend more time/space explaining why is important to investigate disordered eating and eating disorders in athletes. Although I am familiar with the literature, the authors assume a lot of knowledge about this topic from the reader. I suggest explaining why this topic is important before describe the Petrie and Greenleaf (2007; 2012) model.

1) Page 3, line 57: The sentence starting with "Participation in competitive sports has the potential to increase the risk of eating disorders and disordered eating in" do not make sense.  Perhaps the authors mean: in athletes?

2) Page 3, lines 63-64: Please explain briefly the dual-pathway model.

3) Page 3, line 73: Authors should clearly state that they are going to explain all the eight factors/mediators included in the model.

4) Pages 3 and 4, lines 78-83: Why the number of years of sport participation and whether an athlete is currently competing or in his/her off-season are elements that may relate to the development of disordered eating? Other variables might have been chosen. Indeed, later (lines 90-101) authors introduced the number of hours spent training per week as a relevant factor of spot pressure. Please clarify because it is misleading.

5) Page 4, lines 84-85: Please provide examples of lean sports. Again, authors assume a lot of knowledge about this topic from the reader.

6) Page 4, line 86: Please include the bracket after the number 15.

7) Page 4, line 92: Do not use the acronym (NCAA) the first time you mentioned a word.

8) Page 5, line 113: I think authors should include also the coach as a source of societal pressure (along with teammates).

9) Page 6, lines 140-142: Authors should explain deeply the debate pertaining to which type(s) of athletes experience the most body dissatisfaction and whether they experience more dissatisfaction than the general population.

10) Page 6, lines 146-148: The sentence "Research has found that negative affect in athletes, especially constructs such as fear and guilt, influenced bulimic behaviour when tested in conjunction with increased body dissatisfaction, dietary intent, and dietary intent and that certain elements of negative affect " do not make sense. Please clarify.

11) Page 8, line 186: Please include a full stop after the word analysis.

**Methods & Results:**

*General feedback:* The authors should have assessed for the presence of psychological disorders and, specifically, for EDs (and eventually removed participants with EDs) or, at least, include the lack of assessment for psychological disorder within the Limitation section.

Indeed, the range of BMI (Table 1) was comprised between 14.1 and 32.4; both a BMI of 14.1 and 32.4 may be indicative of an ED/disordered eating. Therefore, the etiological model was tested in a sample of athletes where the prevalence of EDs and disordered eating was uncertain. Authors should include this information in the limitation section.

Furthermore, authors should include internal consistency reliabilities for the current study instead of from others' investigations (see Measures section).

1) Page 9, lines 217-219: Why 10 hours of training should be an index of significant involvement in sport activity (instead of a lower/higher number of hours)?

2) Page 9, lines 227-229: It could be useful for readers the inclusion of % for each sport included in the study.

3) Page 10, lines 253-245: It is not clear to me why authors operationalized sport pressure with participation in an individual/team sport. They did not include any reference pertaining to this topic in the introduction.

4) Page 12, line 299: Include a full stop after the question mark.

**Discussion:**

*General feedback:* The discussion largely repeats the findings of the analyses, which is helpful to the reader. However, the authors do not give enough attention to theoretical explanations of the findings (i.e. page 21, lines 466-467: please provide explanations for the invariance of the model across lean and non-lean sports). In general, the authors need to spend less time re-stating their results and more time discussing them. Why are the important? What is the next step?

Furthermore, authors should discuss the clinical implications of the current study. Why your results pertaining to the Petrie and Greenleaf (2007; 2012) model are important in terms of clinical implications?

1) Page 20, line 440: Please include a full stop.

2) Page 20, line 444: Authors should rephrase the discussion in accordance with the results they found. They should refer only to binge eating and bulimia symptoms instead of talking about disordered eating in general. See also page 21, line 488.

3) Page 21: Authors should include in the future directions section the inclusion of athletes younger than 18 yeas old, given that this population was overlook by authors but represents an high-risk population for the development of EDs.

**Figures:**

Please include the number and the legend for each figure.
